# A Systematic Review for Estimation of HTLV-I Infection in the Blood Donors of Iran

**Published:** 2013-03

**Authors:** Mohammad Reza Hedayati-Moghaddam

**Affiliations:** Research Centre for HIV/AIDS, HTLV and Viral Hepatitis, Iranian Academic Centre for Education, Culture & Research (ACECR), Mashhad Branch, Mashhad, Iran

**Keywords:** HTLV-I infection, Systematic review, Meta-analysis, Blood donors, Iran

## Introduction

HTLV-I infection is a worldwide health problem and approximately 15–20 million persons are estimated to be infected with this infection ^(^^1^^)^. High HTLV-I seroprevalence rates in the general population or specific groups such as blood donors, have been reported from southwest of Japan, Caribbean basin, South America, Sub-Saharan Africa, and northeastern Iran ^(^^1^^,^^2^^)^. 

Although most infected people remain asymptomatic, the virus is associated with exceptionally severe diseases, such as adult T-cell leukemia/lymphoma (ATL), and HTLV-I-associated myelopathy/tropical spastic paraparesis (HAM/TSP) ^(^^3^^)^. HTLV-I infection could transmit from mother to child, predominantly through breastfeeding, via sexual intercourse, and parenteral transmission by transfusion of infected cellular blood products or sharing of needles and syringes ^(^^1^^,^^4^^)^.

Presence of HTLV-I infection was reported in 1990 among the Jews emigrated from Mashhad, northeast of Iran ^(^^5^^)^, and then some patients with ATL were indentified in Mashhad who were seropositive for HTLV-I^ (^^6^^)^. In later studies, the rate of HTLV-I infection in Mashhad was reported 3% in general population ^(^^7^^)^ and about 2% in blood donors ^(^^8^^)^ in 1996. There were some small to large scale epidemiological studies that have reported the prevalence of HTLV-I infection in blood donors from various regions of the country. The widest survey has been conducted by Rezvan *et al*. in 21 regional blood centres in 1996. This study reported a 0.29% rate for HTLV-I infection in total serum samples; 1.97% among Mashhadi blood donors, and zero to 0.5 percent in other centres ^(^^8^^)^. Moreover, later surveys in several regions of Iran reported different rates of the infection in blood donors. Nevertheless, there is no overall estimation of the infection in the country. This study conducted to accurately estimate the prevalence of HTLV-I infection in the Iranian blood donors through a comprehensive systematic review of literature and evidences.

## Methods

*Study Question*

The interested outcome was the presence of HTLV-I antibody in blood samples of the Iranian blood donors, based on any blood tests or even if laboratory tests are not identified clearly, until August 2011.

*Search Strategy*

“HTLV” was used as a key word anywhere in the text for searching national electronic databases and websites. “HTLV” anywhere in the text (all fields), and “Iran”, “Iranian” or “Iranians” in the title, subject terms (keywords) or affiliation were also used for searching other databases.

*Electronic Databases*

Seventeen electronic bibliographic databases, and publishers of the health and biological sciences were searched. These included: BioMedCentral, BMJ Journals, Cochrane Library, Directory of Open Access Journals (DOAJ), Ebscohost, Emerald Journals, Google Scholar, MD Consult, OvidSP, Oxford Journals, ProQuest, PubMed, ScienceDirect, Scopus, SpringerLink Contemporary, Web of Knowledge (ISI), and Wiley InterScience Journals. Moreover, all seven Iranian databases of literatures including Iranian Research Institute for Information Science and Technology (IranDoc), Iranian Database of Medical Sciences Papers (IranMedex), Iranian Database of Publication (Magiran), Global Medical Articles Library (Medlib), National Management System for Science and Technology Information (ISNet), the Regional Information Centre for Science & Technology (RICeST), and Scientific Information Database (SID) were investigated. 

*Gray Literature Search*

All of Iranian medical universities’ websites as well as Iranian databases such as IranDoc, ISNet, and RICeST were searched for reports of scientific congresses, research projects, and dissertations. Also libraries of faculties of medicine from Mashhad University of Medical Sciences (MUMS), and Islamic Azad University, Mashhad branch, were investigated for their thesis archives. National reports from Centre for Disease Control (CDC) of the Iranian Ministry of Health, and the Iranian Blood Transfusion Organization (IBTO) websites were also investigated. Finally, cited items of identified studies were screened.

*Critical Appraisal and Selection of Studies*

All titles and abstracts were screened, and relevant citations were reviewed thoroughly and checked for eligibility criteria to include the studies in the analysis. The inclusion criteria were all cross-sectional surveys that have employed appropriate sampling methods, had adequate sample size (more than 200 individuals), and provided estimation of prevalence of HTLV-I infection in blood donors using valid measurement methods in both English and Persian languages.

*Data Extraction*

The selected and included citations were reviewed, and the findings were extracted to a sheet. The extracted data was first author, year of the study, province/city of the study, sampling method, sample size, name of the kits and methods used for HTLV antibody detection, mean and standard deviation of subjects’ age, percentage of male subjects, and total and sex-related HTLV-I point prevalence.

*Statistical Analysis*

The total and sex-related point prevalence of HTLV-I infection was recalculated according to the number of infected cases, and total observations reported in each study. Binomial 95% CI was computed using EPI 6.0 software (CDC, USA). Meta-analysis method using random effect model was used based on the results of heterogeneity test (Cochrane Q) with significance set at *P<* 0.05. 

## Results

*Search Result*

175 relevant nonduplicate citations of all 843 electronic searched citations were found, of which 17 studies discussed seroprevalence of HTLV-I in Iranian blood donors ^(^^8^^-^^24^^)^. Two studies were excluded due to overlapping samples ^(^^12^^,^^15^^).^

**Figure 1:**
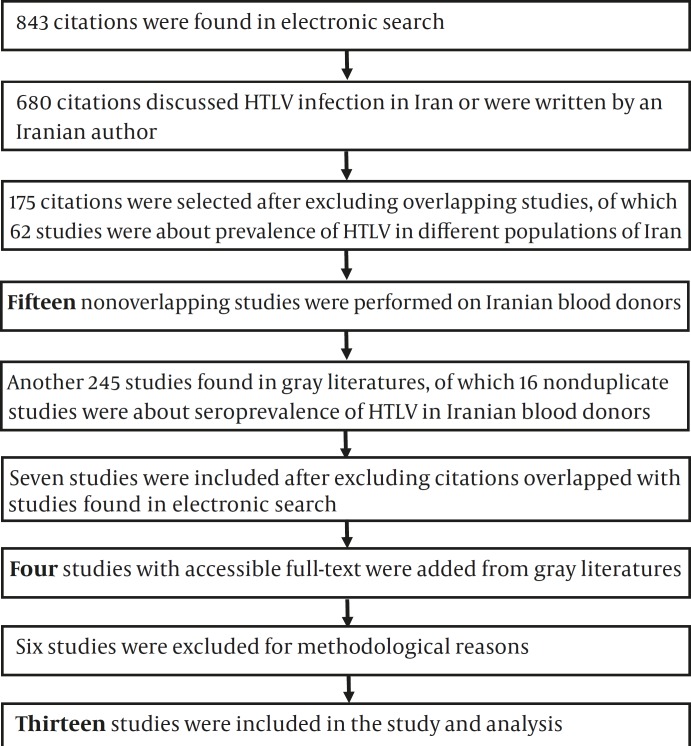
Diagram of Searches and Systematic Review for Prevalence of HTLV-I Infection in Iranian Blood Donors

In gray literature search another 245 surveys including 104 published in abstract books of congresses, 64 research reports, and 77 theses were found, of which seven nonoverlapping studies were with subjects in blood donors ^(^^25^^-^^31^^)^. Access to data of three studies was not possible in spite of requesting from related institutes ^(^^29^^-^^31^^)^. Moreover, one relevant study published in Persian found from backward citations ^(^^32^^)^ but had overlap with another English paper ^(^^8^^)^. On the other hand, no relevant data was found in searching organizations reports. After excluding studies with samples that were not representative of the target population ^(^^11^^,^^18^^, ^^24^^)^ or with inadequate samples size ^(^^13^^, ^^28^^)^ and/or studies which did not use confirmatory tests ^(^^13^^,^^16^^)^, finally 13 studies were selected. The detailed search process is demonstrated in Figure 1.

*Studies*

Thirteen relevant studies with a total of 1,091,361 subjects were found of satisfactory quality ^(^^8^^-^^10^^,^^14^^,^^17^^, ^^19^^-^^23^^, ^^25^^-^^27^^)^. All included surveys were cross sectional studies conducted on Iranian blood donors from 1996 to 2009 with sample size ranging from 960 to 243,856 (Table 1). One study had been conducted in the regional blood centres of 21cities in the country ^(^^8^^)^ and six were from Mashhad covering the years from 1997 to 2000 ^(^^9^^,^^14^^)^ and 2004 to 2009 ^(^^10^^,^^22^^,^^25^^,^^26^^)^. 

The age of the subjects was only determined in five studies ^(^^9^^,^^10^^,^^19^^,^^20^^,^^27^^)^ which was between 17 and 65. Sex distribution of subjects was reported in nine studies ^(^^9^^,^^10^^, ^^17^^,^^19^^,^^20^^,^^22^^,^^23^^,^^25^^,^^27^^)^, 79 to 94 percent of the subjects were males. All studies had used ELISA tests by several HTLV detection kits (such as Biomeriux, Diapro, Genlab, MP Diagnostics, Organon, Ortho, and ZeptoMetrix) for primary screening, and western blot by some HTLV-blot kits, mainly Genlab, with or without PCR as confirmatory tests.

*HTLV-I Infection Prevalence*

Mashhad, the capital city of Razavi Khorasan province, has still remained as the most prevalent area for HTLV-I infection; however, the infection rate has fallen from 1.16% in 1997-2000 ^(^^14^^) ^to 0.38% in 2008-2009 ^(^^22^^)^.Urmia, the capital city of West Azerbaijan province, has the second place, and HTLV-I infection prevalence has reported as high as 0.34% ^(^^19^^)^. Prevalence of the infection among blood donation volunteers in Karaj, the capital city of Alborz province, and Hormozgan and Ilam provinces ranges from 0.11 to 0.21 percent ^(^^20^^,^^21^^,^^27^^)^. The lowest prevalence rates were seen in south Khorasan (0.042%) ^(^^23^^)^ and Bushehr (0.013%)^ (^^17^^)^. Prevalence of the infection according to the sex was verified or calculable only in six surveys. Except for one study ^(^^17^^)^, the infection rate in female blood donors was 2 to 6 times higher than males (Table 1). 

**Figure 2:**
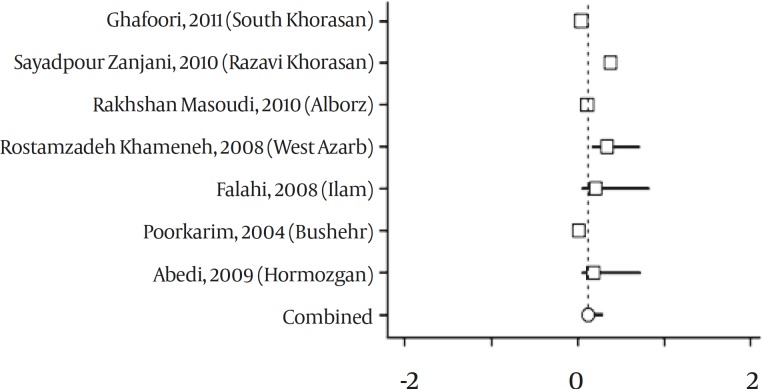
Forest Plot of Surveys on Prevalence of HTLV-I Infection in Iranian Blood Donors

**Figure 3:**
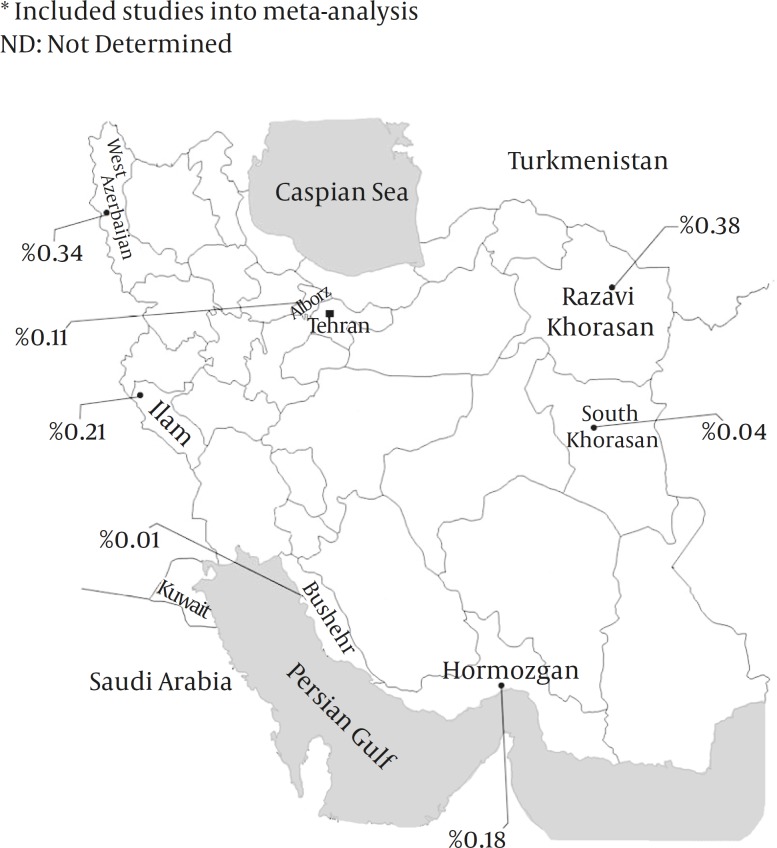
Prevalence of HTLV-I Infection Among Blood Donors in Different Provinces of Iran

From six studies conducted in Mashhad, the latest survey included in meta-analysis ^(^^17^^)^ and a study performed in 21 provinces was excluded ^(^^8^^)^. According to data from seven studies, point estimation for HTLV prevalence in Iranian blood donors was 0.119% (95% CI: 0.050-0.287 percent). (Figures 2 & 3). By using heterogeneity test, a significant variation was found between the studies (Q= 151.13, df=6, *P<*0.001, I-squared= 96.03%). 

**Table 1:** HTLV-I Infection Prevalence Rate in Iranian Blood Donors

Country region	Province	Time of study	Sample Size	Total prevalence; % (95% CI)	Prevalence in men; % (95% CI)	Prevalence in women; % (95% CI)
Total	21 provinces from the country (8)	1996	15,866	0.296 (0.218-0.394)	ND	ND
East	South Khorasan (23)*	2006-9	42,652	0.042 (0.025-0.067)	ND	ND
Northeast	Razavi Khorasan (9)	1999	28,487	0.769 (0.671-0.877)	ND	ND
	Razavi Khorasan (14)	1997-2000	184,496	1.161 (1.113-1.211)	ND	ND
	Razavi Khorasan (10)	2004-6	232,648	0.453 (0.426-0.481)	0.422 (0.395-0.450)	0.762 (0.650-0.888)
	Razavi Khorasan (25)	2006-8	243,856	0.406 (0.381-0.432 0	0.362 (0.338-0.388)	0.980 (0.839-1.138)
	Razavi Khorasan (26)	2006-8	201,719	0.421 (0.394-0.451)	ND	ND
	Razavi Khorasan (22)*	2008-9	79,687	0.378 (0.336-0.423)	ND	ND
Centre	Alborz (27)*	2009	32,958	0.112 (0.79-0.155)	0.103 (0.071-0.146)	0.257 (0.084-0.599)
Northwest	West Azarbaijan (19)*	2005-6	2046	0.342 (0.138-0.704)	0.262 (0.085-0.610)	1.471 (0.179-5.211)
West	Ilam (20)*	2006-7	960	0.208 (0.025-0.751)	0.122 (0-0.676)	0.725 (0.018-3.971)
Southwest	Bushehr (17)*	2002-3	22,740	0.013 (0-0.039)	0.017 (0-0.049)	0 (0-0.078)
South	Hormozgan (21)*	2007-8	1100	0.182 (0.022-0.655)	ND	ND

* Included studies into meta-analysis

ND: Not Determined

## Discussion

This review for estimating the overall prevalence of HTLV-I infection in Iranian blood donors indicated that distribution of HTLV-I infection in our country is not uniform, and there were significant discrepancies in different provinces regarding the infection rates. Mashhad in northeast of Iran, as previously reported ^(^^7^^, ^^8^^, ^^11^^)^, has the highest frequency of the infection. Our recent study showed that Mashhad has still remained an endemic area for HTLV-I infection with 2.12% prevalence in the general population^ (^^33^^)^. Also high prevalence of the infection in other cities of Razavi Khorasan province such as Neyshabour, and Sabzevar has been demonstrated ^(^^34^^, ^^35^^)^. Nevertheless, a declining trend in the infection prevalence among blood donors in Mashhad has been occurred. This decline could be attributed to an important strategy for donor screening. Since 1995, all donated blood samples in the blood transfusion centre of North, Razavi, and South Khorasan provinces, are routinely screened for HTLV-I ^(^^36^^)^. Similarly, a signiﬁcant decline of the carrier rate among younger blood donors has been reported in Japan, due to screening blood donors for HTLV-I, and refraining from breastfeeding ^(^^37^^)^. 

Dissimilar distribution of HTLV-I infection in different parts of Iran might be due to variant demographic characteristics of studied population or different applied laboratory kits. However, most blood donors (92%) in Iran are men, and according to the Iranian Blood Transfusion Organization (IBTO) criteria the age range of donors must be between 18 and 65 years ^(^^38^^)^. On the other hand, geographical clustering of the virus among neighbours has to be considered as important issue ^(^^1^^,^^3^^)^. In Turkmenistan, in proximity of Northeastern Iran, 0.2% of blood donors were HTLV-I infected ^(^^39^^)^. In contrast, the infection is probably rare in other neighboring countries of Iran. In Kuwait and Saudi Arabia, in proximity of Southwestern Iran, 0.016% and 0.046% of national blood donors showed antibodies against HTLV-I, respectively ^(^^40^^,^^41^^)^. 

Also this review showed that HTLV-I infection is not probably limited to the northeast of Iran. It seems that strict screening of donated bloods should be considered in other provinces such as West Azarbaijan where evidences suggest relatively considerable rate of the infection. 
